# Long non-coding RNA-PCGEM1 contributes to prostate cancer progression by sponging microRNA miR-129-5p to enhance chromatin licensing and DNA replication factor 1 expression

**DOI:** 10.1080/21655979.2022.2059936

**Published:** 2022-04-12

**Authors:** Qiao Fu, Fangfang Wang, Jun Yang, Wei Sun, Zhi Hu, Lv Xu, Hao Chu, Xiao Wang, Wei Zhang

**Affiliations:** Department of Urology, The Third Hospital of Wuhan, Wuhan Hubei, China

**Keywords:** lncRNA-PCGEM1, prostate cancer, PCa, miR-129-5p, CDT1

## Abstract

PCGEM1 facilitates prostate cancer (PCa) progression. This study aimed to elucidate the mechanism of action of PCGEM1 in PCa. The expression of PCGEM1, microRNA miR-129-5p, chromatin licensing, and DNA replication factor 1 (CDT1) was detected by quantitative reverse transcription-PCR (qRT-PCR). A series of function experiments including cell counting kit-8 (CCK-8), caspase-3 activity, and cell cycle assays were performed to evaluate the influence of PCGEM1, miR-129-5p, and CDT1 on the biological processes of PCa cells. CyclinD1, cyclin dependent kinase 4 (CDK4), Bax, and Bcl-2 protein levels were measured by western blotting. Subcellular isolation revealed the distribution of PCa cells. The connections between PCGEM1, miR-129-5p, and CDT1 were evaluated by luciferase, RIP assay, and Pearson correlation analysis. Both PCGEM1 and CDT1 were upregulated in PCa, while miR-129-5p was downregulated and negatively correlated with PCGEM1 and CDT1. Downregulation of PCGEM1 or CDT1 inhibited the viability, promoted apoptosis and cycle arrest of PCa cells *in vitro*, and controlled tumor growth *in vivo*. PCGEM1 plays a crucial role in the progression of PCa by sponging miR-129-5p as a ceRNA of CDT1. PCGEM1 is a CDT1-dependent PCa promoter site that absorbs miR-129-5p.

## GRAPHICAL ABSTRACT

**Figure:**
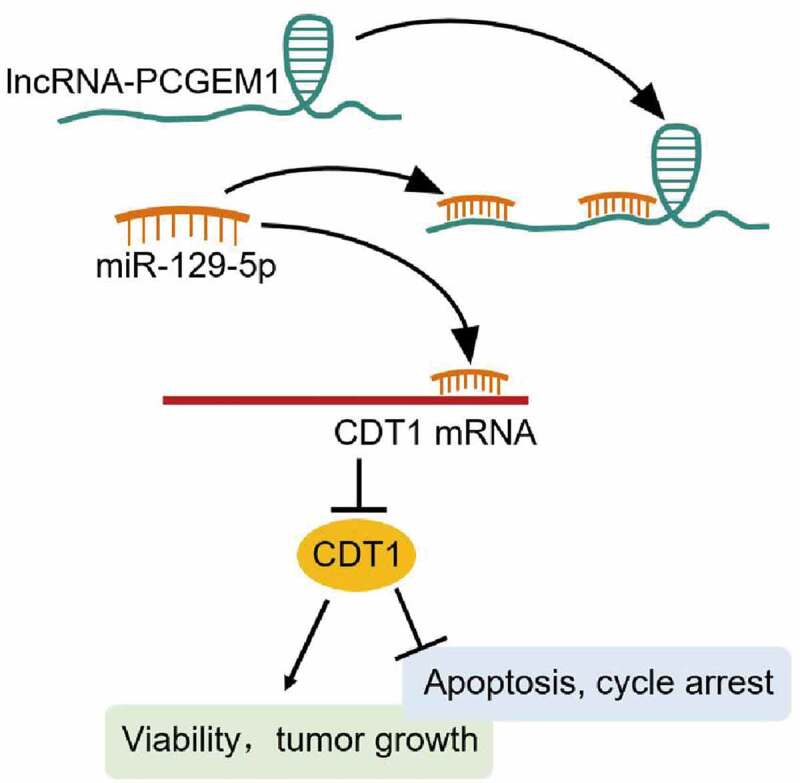

## Introduction

There were more than 1.2 million new cases of prostate cancer (PCa) in 2018, resulting in approximately 300,000 deaths, which accounted for 3.8% of all cancer-related deaths in men [1,2]. The diversity of PCa monitoring methods and the asymptomatic nature of the early stages of cancer make early detection extremely difficult [3]. Furthermore, alterations in genetic composition have been shown to significantly increase the risk of family PCa worldwide [4]. Therefore, there is an urgent need to identify PCa risk genes and develop effective screening and treatment methods.

Long non-coding RNAs (lncRNAs), transcripts larger than 200 bp, are a class of RNAs that do not translate into proteins [5]. Accumulating evidence suggests that lncRNAs participate in the regulation of gene expression in the cytoplasm and nucleus through a variety of pathways. In addition, differential expression of lncRNA has been found to play a critical role in tumors, including PCa [6]. PCGEM1 is highly prostate-specific and attractive biomarker [7]. Several studies have shown that PCGEM1 is upregulated in PCa tissues, indicating malignant progression of tumors [8,9]. Nevertheless, the mechanism of PCGEM1 in PCa remains unknown.

MicroRNAs participate in a variety of physiological processes that influence cancer occurrence through complex cross-regulation between lncRNAs and mRNAs [10]. As a key inhibitor involved in the occurrence of various cancers, miR-129-5p is expressed at low levels in the lung [11], colon [12], and PCa [13]. Previous studies have shown that miR-129-5p serves as a downstream target of PCGEM1, regulating the occurrence and development of gastric cancer [14] and endometrial cancer [15]. However, the potential role of PCGEM1/miR-129-5p in PCa progression has not yet been studied.

Chromatin licensing and DNA replication factor 1 (CDT1), located at the centromere in an Ndc80 complex-dependent manner, has an independent mitotic role in addition to its prototypic function in the origin licensing of DNA replication [16]. Overexpression of CDT1 was revealed to be a predictor of low survival in hepatocellular carcinoma patients [17] and a prognostic marker for breast cancer patients [18]. Importantly, abnormal expression of CDT1 was reported to predict the initiation risk of DNA replication in PCa [19]. However, the function and mechanism of CDT1 in PCa have not yet been explored.

Herein, the specific relationship between PCGEM1/miR-129-5p/CDT1 in PCa was verified, and its effect on cancer cell biological function was discussed. We hypothesized that PCGEM1 is upregulated in PCa, promoting the malignant behavior of PCa by sponging miR-129-5p and releasing CDT1. The aim of this study was to develop an effective PCa screening and treatment strategy.

## Methods

### Tissue collection

All studies involving patient samples were approved by the ethics committee of the Third Hospital of Wuhan (approval number: 武三医伦 KY2022-012). PCa tissue and adjacent normal tissue from 26 patients with radical prostatectomy excised PCa were immediately frozen at −80°C. All patients voluntarily participated in the study and provided written informed consent. None of the patients received neoadjuvant chemotherapy or endocrine therapy before surgery. The clinical characteristics of the patients with PCa are listed in Supplementary Table 1.

### Cell Culture

The human prostate cell line (LNCAP, 22RV1, and MDA-PCA-2B) and human prostate epithelial cell line RWPE1 were obtained from ATCC (USA). RWPE-1 was stored in K-SFM medium (Gibco, USA). 22RV1 and LNCaP cells were stored in DMEM, and MDA-PCA-2B cells were cultured in F-12 K medium. All cell lines were supplemented with 1% P/S (Sangon, China) and 10% fetal bovine serum (FBS, Gibco) and placed in an incubator at 37°C and 5% CO_2_.

### Nuclear cytoplasmic fractionation

The position of PCGEM1 in the cells was mapped using nuclear cytoplasmic separation. NE PER Nuclear and Cytoplasmic Extraction Reagents (Thermo Fisher Scientific) were used to isolate the nucleus and cytoplasm of LNCaP and 22RV1 cells. Nuclear control transcript (Uracil6, U6), cytoplasmic control transcript (glyceraldehyde-3-phosphate dehydrogenase, GAPDH), and PCGEM1 levels in the nucleus and cytoplasm were determined by qRT-PCR [20].

### Cell transfection

GenePharma (China) provided siRNA for PCGEM1 or CDT1 (si-PCGEM1 or si-CDT1) and its negative control siRNA(si-NC). MiR-129-5p inhibitor and inhibitor NC were collected from Switchgear Genomics (USA). LNCaP and 22RV1 cells were seeded into 6-well plates (5 × 10^4^ cells/mL) and incubated overnight. Lipofectamine2000 (Thermo Fisher Scientific, USA) was used to transfect 50 nM siRNA or 100 nM inhibitor into cells, which were collected after 48 h transfection at 37°C. The transfection efficiency was then determined.

### Quantitative reverse transcription-PCR (qRT-PCR) assay

RNA was extracted from whole-cell lysates using the Eastep Super RNA Extract Reagent Kit (Promega, USA). The first-strand cDNA was inverted using a cDNA synthesis kit (Invitrogen). SYBR qPCR Master Mix (Vazyme, USA) was used for PCR in the Prism 7000 Real-Time PCR system, with GAPDH serving as the internal standard. In addition, the NucleoSpin® miRNA kit (Macherey Nagel, France) was used to extract miRNAs. Small nuclear U6B (RNU6B) RNA was used as a housekeeping gene. cDNA synthesis was performed using an miRScript II RT kit. miRNAs were quantified using the Miscript SYBR Green PCR Kit. mRNA and miRNA expression was measured by 2^−ΔΔCt^ [21]. The primer sequences are listed in Table 1.

**Table 1.** The sequences of the primers in this study

Primer	Sequences	Product length
**CDKN2A**	Forward: 5′-TGGAGTCCGTCCTTCCAATG-3′	164 bp
	Reverse: 5′-GCGTGTAAAACGGCTGTCTG-3′	
**PTK6**	Forward: 5′-GCTATGTGCCCCACAACTACC-3′	401 bp
	Reverse: 5′-CCTGCAGAGCGTGAACTCC-3′	
**TK1**	Forward: 5′-CTTGGCCTTCTGGGAACTCT-3′	67 bp
	Reverse: 5′-GGTAGGAGAGGAGGGAGCAT-3′	
**CDT1**	Forward: 5′-GACATGATGCGTAGGCGTTTT-3′	134 bp
	Reverse: 5′-GAGCTGGTAATCTGACCTCCT-3′	
**PCGEM1**	Forward: 5′-CTGTGTCTGCAACTTCCTCTAA-3′	87 bp
	Reverse: 5′-TCCCAGTGCATCTCGTAGTA-3′	
**miR-129-5p**	Forward: 5’-GGGGGTTTTTGCGGTCTGG-3’	43 bp
	Reverse: 5’-AGTGCGTGTCGTGGAGTC-3’	
**GAPDH**	Forward: 5’-TCAACGACCACTTTGTCAAGCTCAGCT-3’	116 bp
	Reverse: 5’-GGTGGTCCAGGGGTCTTAC-3’	
**U6**	Forward: 5’-CTCGCTTCGGCAGCACA-3’	95 bp
	Reverse: 5’-AACGCTTCACGAATTTGCGT-3’	

### Cell counting kit-8 (CCK-8) assay

A CCK-8 kit (Roche, Basel, Switzerland) was used to measure cell viability. Briefly, 22RV1 and LNCaP cells were seeded into 96-well plates (3 × 10^3^ cells per well). After transfection for 24, 48, 72, and 96 h, 10 μL CCK-8 solution was added to each well at 37°C for 2 h. Absorbance was measured at 450 nm using an FLx800 fluorescence microplate reader (BioTek, USA) [22].

### Caspase-3 activity assay

The apoptosis of LNCaP and 22RV1 cells was detected using a caspase-3 activity assay kit (Beyotime, China). LNCaP and 22RV1 cells (1 × 10^5^) were lysed in cell lysis buffer for 15 min at 4°C, and protein concentration was determined using Pierce bicinchoninic acid (BCA) (Thermo Scientific). After incubating the reaction mixture system composed of cell lysate (10 μL), detection buffer solution (80 μL), and Ac-DEVD-pNA (10 μL, 2 mM) in 96-well plates at 37°C for 2 h, the optical density was measured using a microplate reader at 405 nm [23].

### Cell cycle assay

After transfection for 72 h, LNCaP and 22RV1 cells (1 × 10^6^ cells) were collected, centrifuged, washed twice with phosphate-buffered saline (PBS), and precipitated. The precipitates were fixed in 70% ethanol at 4°C for 4 h. Next, the cells were stained with 500 µL of staining solution (staining buffer 465 µL, 50× RNase A 10 µL, 20× propidium iodide 25 µL) at 37°C for 1 h according to the manufacturer’s instructions. Finally, the cell cycle distribution was determined using flow cytometry (FACS CantoTM II Flow Cytometer, BD Biosciences, USA) [24].

### Tumor growth assay

The Shanghai Experimental Animal Center (China) provided BALB/c nude mice (aged 5 weeks, two mice). LNCaP cells (5 × 10^6^/mL) treated with PCGEM1 shRNA or sh-NC (GenePharma, China) were subcutaneously injected into the right side of the posterior flank of mice, and the mice were divided into sh-lnc (n = 5) and sh-NC groups (n = 5). Tumor size was measured with calipers every week. After feeding for 5 weeks, nude mice were euthanized with excessive CO_2_, and tumors were removed using a scalpel, photographed, and weighed. The animal procedures were approved by our animal committee [25].

### Luciferase assay

The 3’-untranslated region (UTR) products of PCGEM1 and CDT1 containing miR-129-5p target sequences were obtained by PCR and delivered into pmirGLO (Promega) to construct wild-type PCGEM1 and CDT1 reporter vectors (PCGEM1-WT and CDT1-WT). Similarly, PCGEM1 (PCGEM1-MUT) and CDT1 mutant vectors (MUT1, MUT2, and Co-MUT) were generated by site-directed mutagenesis. LNCaP and 22RV1 cells cultured for 24 h were co-transfected with wild-type or mutant vectors and miR-129-5p mimic for 48 h, using Lipofectamine 2000 (Invitrogen). Luciferase activity was analyzed using a dual-luciferase reporter assay system (Promega) [26].

### RNA immunoprecipitation (RIP) assay

The BersinBio^TM^ RIP kit (Ca# Bes5101, BersinBio, China) was used for RIP, following the manufacturer’s instructions. Briefly, LNCaP and 22RV1 cells (1 × 10^7^ cells) were lysed using RNA lysis buffer for 20 min on ice. Cell lysates were incubated with DNase for 10 min at 37°C and then centrifuged at 16,100 *g* for 10 min. Then, samples were conjugated with magnetic beads with 5 μg Ago2 or negative control IgG antibody and incubated at 4°C for 4 h. Then, 40 μL protein A-Sepharose was added to each sample, and the mixture was incubated at 4°C for 4 h. After washing with PBS, the precipitated RNA was resuspended and analyzed by qRT-PCR [27].

### Western blot analysis

LNCaP and 22RV1 cells were treated with RIPA buffer (Beyotime) to obtain proteins, which were quantified using BCA (Pierce, USA). A 10% sulfate-polyacrylamide gel electrophoresis was utilized to separate the equivalent amount of protein, which was then delivered to a polyvinylidene fluoride membrane. The membrane was sealed with PBST containing 5% skim milk, and then coexisted with CDT1 (ab202067; Abcam, UK), Bax (ab3191; Abcam), Bcl-2 (ab218123; Abcam), CyclinD1 (ab134175; Abcam), CDK4 (ab68266; Abcam), or GAPDH antibody (ab181602; Abcam) at 4°C overnight. The membrane was incubated with the secondary antibody (ab205718; Abcam) for 1 h at 37°C. Protein bands were observed using enhanced chemiluminescence (ECL) substrates (Pierce, USA) [28].

### Statistical analysis

Data are expressed as the mean ± standard deviation and were analyzed using SPSS Statistics 21 software (SPSS, USA). Statistical significance was set at P < 0.05. Statistical significance was analyzed using one-way analysis of variance (ANOVA) or Student’s *t*-test.

### Results

Herein, we illustrated the regulatory network of PCGEM1/miR-129-5p/CDT1 in PCa and hypothesized that PCGEM1promoted malignant behavior of PCa cells by sponging miR-129-5p and releasing CDT1. Clinical specimen analysis showed that both PCGEM1 and CDT1 were upregulated, while miR-129-5p was downregulated in PCa. In addition, loss-of-function analysis revealed that PCGEM1 or CDT1 knockdown inhibited viability and tumor growth and promoted apoptosis and cycle arrest in PCa cells. PCGEM1 is a CDT1-dependent PCa promoter site that absorbs miR-129-5p.

## PCGEM1/miR-129-5p/CDT1 axis was confirmed in PCa

**Figure 1.**
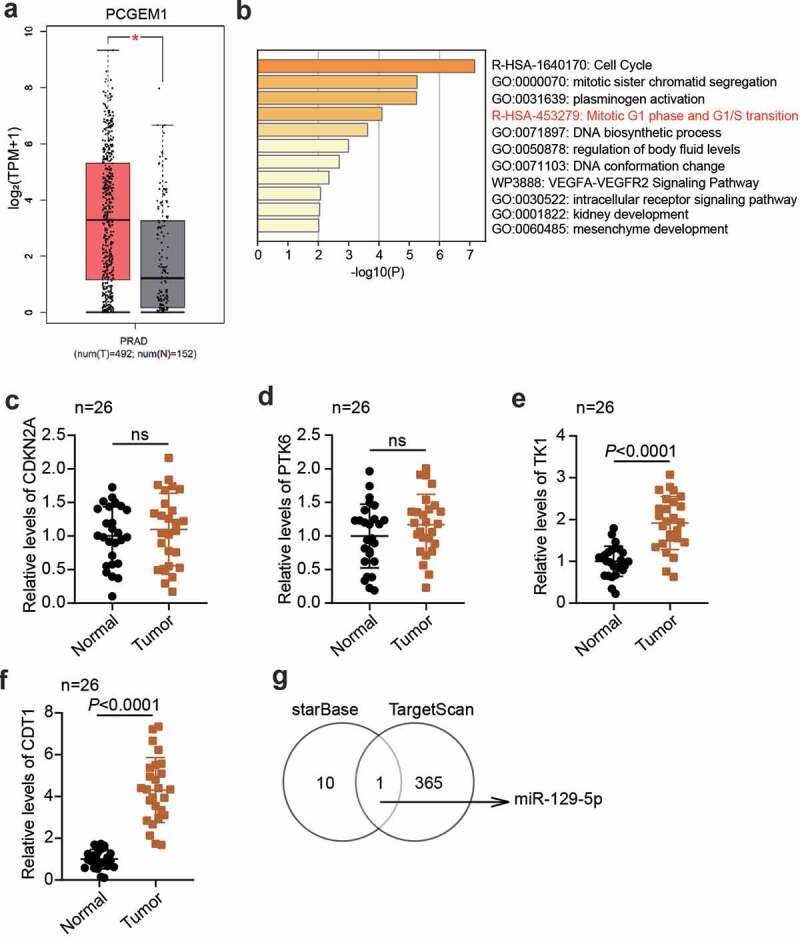
**The key regulators were confirmed in PCa**. (a) the PCGEM1 expression in PCa was confirmed by GEPIA database. (b) the key biological process involving the key genes in GSE38241 was enriched by metascape. (c-f) analysis of CDKN2A (c), PTK6 (d), TK1 (e) and CDT1 (f)level in PCa as well as normal samples based on qRT-PCR assay. (n = 26). (g) miR-129-5p was the only miRNA binding to PCGEM1 and CDT1 based on the prediction of starBase and TargetScan.

According to the data from GEPIA, PCGEM1 was upregulated in PCa (Figure 1a). GSE38241 was the mRNA expression profile including PCa samples and non-cancer samples, which was utilized to screen 63 upregulated genes with adj.P < 0.01 and logFC>2. Then, 63 genes were uploaded to Metascape for biological analysis, showing that four genes (CDKN2A, PTK6, TK1, and CDT1) were related to the mitotic G1 phase and G1/S transition (Figure 1b). Next, qRT-PCR was used to detect differences between these four genes in PCa tissues and normal tissues, and it was found that CDT1 was the most upregulated mRNA in cancer tissues (Figure 1c,d,e,f). Therefore, CDT1was selected as an interesting mRNA. To identify key miRNAs, starBase was performed to identify the downstream miRNAs of PCGEM1, and TargetScan was used to predict the upstream miRNAs of CDT1. Finally, miR-129-5p was confirmed to be able to bind to PCGEM1 and CDT1 (Figure 1g).

## PCGEM1 was up-regulated in PCa and affected the malignant phenotypes of cancer cells

**Figure 2.**
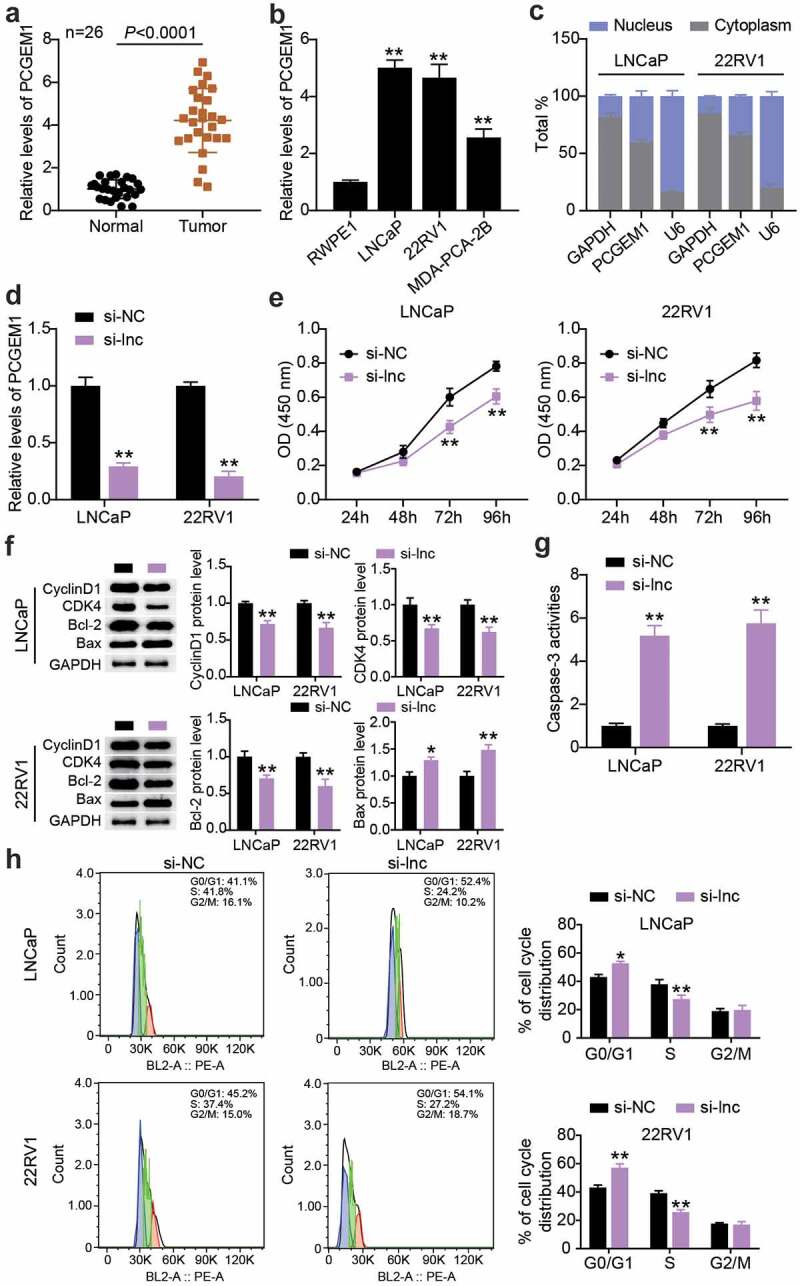
**PCGEM1 was up-regulated in PCa and affected the malignant phenotypes of cancer cells**. (a) analysis of PCGEM1 expression in PCa as well as normal samples based on qRT-PCR. (b) PCGEM1 level in prostate cell lines was uncovered using qRT-PCR. **P < 0.001 vs. RWPE1. (c) Subcellular fractionation for PCGEM1 in 22RV1 and LNCaP cells. (d) knockdown efficiency of PCGEM1 was evaluated utilizing qRT-PCR assay. (e) viability of LNCaP and 22RV1 cells treated with si-lnc was uncovered utilizing CCK-8. (f) bax, Bcl-2, CyclinD1 and CDK4 protein levels were evaluated using western blotting assay in LNCaP and 22RV1 cells treated by si-lnc. (g) caspase-3 activity of LNCaP and 22RV1 cells delivered si-lnc were assessed using the caspase-3 activity assay. (h) Cell cycle of LNCaP as well as 22RV1 delivered si-lnc was assessed using flow cytometry assay. *P < 0.05; **P < 0.001 vs. si-NC.

We determined PCGEM1 expression in PCa and normal tissues. The results showed that PCGEM1 levels in tumor tissues were approximately 4-fold higher than those in normal tissues (Figure 2a). PCGEM1 expression was detected in the three PCa cells. As illustrated in (Figure 2b), the expression of PCGEM1 in LNCaP, 22RV1, and MDA-PCA-2B cells was 5, 4.6, and 2.5 times higher than that in RWPE1 cells, respectively. The nucleoplasmic regionalization of PCGEM1 was analyzed using the subcellular classification of LNCaP and 22RV1 cells. We found that PCGEM1 expression was higher in the cytoplasm than in the nucleus, indicating that PCGEM1 is mainly localized in the cytoplasm and may play a role through transcription (Figure 2c). Subsequently, this study clarified PCGEM1ʹs role in the function of PCa cells. First, 22RV1 and LNCaP cells were treated with siRNA to downregulate PCGEM1 expression, which showed that the PCGEM1 level in the si-lnc group was 20% of that in the si-NC group (Figure 2d). Second, in *in vitro* experiments, CCK-8 analysis revealed that inhibition of PCGEM1 reduced cell viability by approximately 25% (Figure 2e). In addition, western blotting revealed down-regulation of CyclinD1, CDK4, and Bcl-2 protein levels and upregulation of Bax protein levels in the si-lnc group compared to the si-NC group (Figure 2f). Caspase-3 activity analysis revealed that the apoptosis rates of LNCaP and 22RV1 cells in the si-lnc group were increased by about 5 and 5.8 times, respectively, compared with those in the si-NC group (Figure 2g). In addition, PCGEM1 silencing enhanced the proportion of G0/G1 phase cells and decreased the proportion of S phase cells (Figure 2h).

## Interference with PCGEM1 inhibited the growth of PCa cells *in vivo*

**Figure 3.**
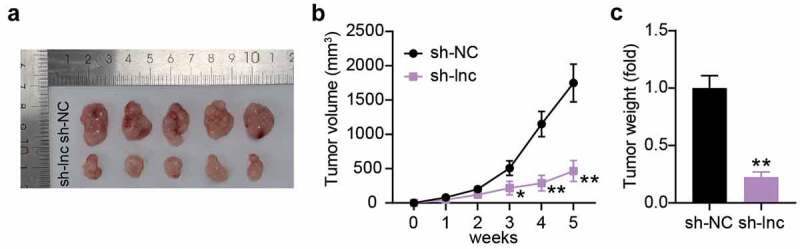
**Interference with PCGEM1 inhibited the growth of PCa cells in vivo**. (a) images of tumor in si-lnc group. (b) the difference of the growth curve of tumor volume in si-lnc groups were portrayed. (c) the difference of tumor weight in si-lnc groups were portrayed. *P < 0.05; **P < 0.001 vs. sh-NC.

To clarify the impact of PCGEM1 on PCa cell growth *in vivo*, we injected LNCaP cells with PCGEM1 knockdown into nude mice. In contrast to the sh-NC group, the tumor morphology and volume of nude mice in the low PCGEM1 expression group were smaller (Figure 3a,b). The tumor was weighed, showing that PCGEM1 silencing attenuated the tumor weight. These results suggest that the knockdown of PCGEM1 may inhibit PCa cell growth *in vivo* (Figure 3c).

## PCGEM1 sponged miR-129-5p

**Figure 4.**
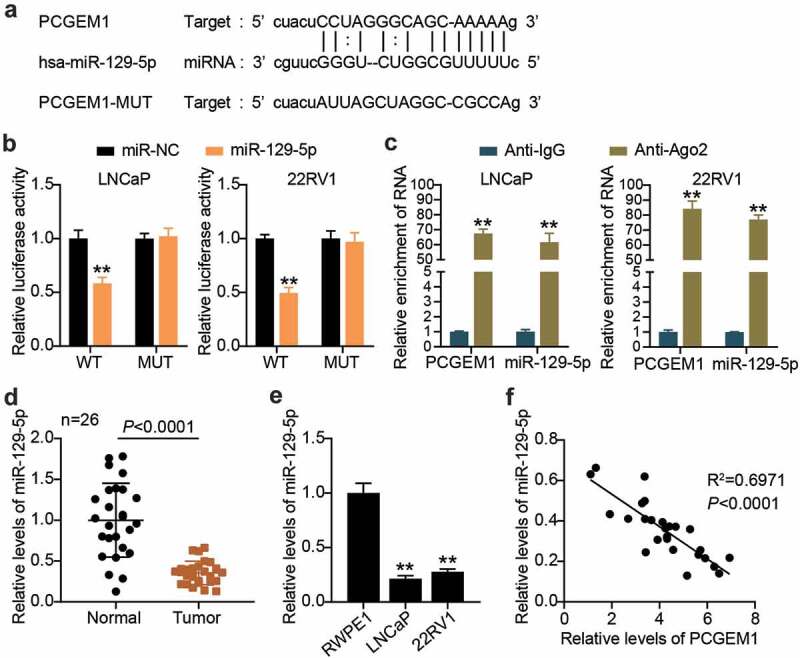
PCGEM1 sponged miR-129-5p. (a) the wild-type and mutant binding site of PCGEM1 for miR-129-5p were constructed. (b) interaction of PCGEM1 and miR-129-5p was ascertained through luciferase assay. **P < 0.001 vs. miR-NC. (c) Relationship of PCGEM1 and miR-129-5p was validated using RIP assays. **P < 0.001 vs. Anti-IgG. (d) Analysis of miR-129-5p level in PCa as well as normal samples based on qRT-PCR. (e) miR-129-5p expression in prostate cell lines was uncovered utilizing qRT-PCR. **P < 0.001 vs. RWPE1. (f) Pearson was utilized for exploring the connection of PCGEM1 with miR-129-5p.

Using bioinformatics analysis, we found that there were binding sites between the pCGEM1 and miR-129-5p sequences (Figure 4a). Next, the binding relationship between pCGEM1 and miR-129-5p was further explored. Luciferase assay results showed that the miR-129-5p ectopic expression only reduced PCGEM1-WT luciferase activity, whereas the luciferase activity of PCGEM1-MUT did not respond to the overexpression of miR-129-5p (Figure 4b). In addition, it was revealed that miR-129-5p and PCGEM1 were enriched in compounds precipitated by anti-Ago2 antibodies in the RIP experiment (Figure 4c). The interaction between PCGEM1 and miR-129-5p has been demonstrated. By measuring miR-129-5p levels in clinical tissues, it was determined that miR-129-5p levels were lower in cancer tissues than in paracancerous specimens (Figure 4d). Similarly, we also found that miR-129-5p expression was significantly lower in both types of cancer cells compared with that in RWPE1 cells (Figure 4e). Pearson correlation analysis also confirmed a negative correlation between PCGEM1 and miR-129-5p in cancer (figure 4f). In conclusion, the PCGEM1 sponge absorbed miR-129-5p.

## PCGEM1/miR-129-5p accelerated the development of PCa

**Figure 5.**
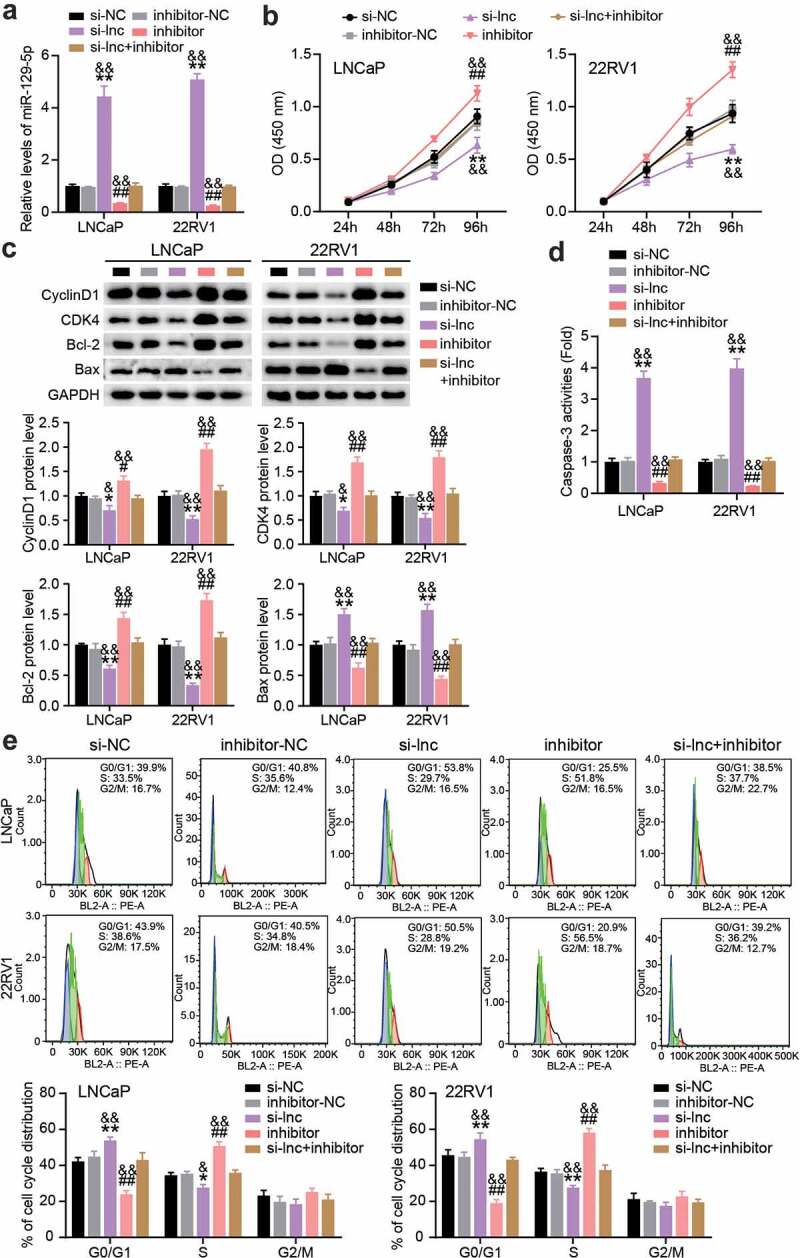
**PCGEM1/miR-129-5p accelerated the development of PCa**. (a) The miR-129-5p level was uncovered utilizing qRT-PCR assay in LNCaP and 22RV1 delivered si-lnc or inhibitor. (b) The viability of LNCaP as well as 22RV1 treated by si-lnc or miR-129-5p inhibitor was uncovered utilizing the CCK-8 assay. (c) Bax, Bcl-2, CyclinD1 and CDK4 protein levels were evaluated using western blotting assay in LNCaP and 22RV1 cells treated by si-lnc or miR-129-5p inhibitor. (d) The caspase-3 activity of LNCaP and 22RV1 cells treated with si-lnc or miR-129-5p inhibitor was assessed using the caspase-3 activity assay. (e) Cell cycle of LNCaP as well as 22RV1 delivered by si-lnc as well as miR-129-5p inhibitor were uncovered utilizing flow cytometry assay. *P < 0.05, **P < 0.001 vs. si-NC; #P < 0.05, ##P < 0.001 vs. inhibitor-NC; &P < 0.05, &&P < 0.001 vs. si-lnc+inhibitor.

In a follow-up study, we performed a rescue trial to verify the role of PCGEM1/miR-129-5p in PCa. First, we reduced the expression of PCGEM1 and miR-129-5p in LNCaP and 22RV1 cells, indicating that miR-129-5p increased after PCGEM1 knockdown, but decreased when miR-129-5p was knocked down (Figure 5a). In functional analysis, as shown in (Figure 5b), CCK-8 showed that cell viability was enhanced by miR-129-5p interference, and miR-129-5p interference counteracted PCGEM1 silencing on cell viability inhibition. In addition, western blotting analysis showed that the protein levels of CyclinD1, CDK4, and Bcl-2 were upregulated and the protein level of Bax was downregulated after miR-129-5p interference, resulting in a reversal of protein levels after PCGEM1 silencing (Figure 5c). Furthermore, miR-129-5p inhibitor inhibited the caspase-3 activity of cancer cells and reversed the enhanced apoptosis effect of si-lnc (Figure 5d). Moreover, flow cytometry revealed that the PCGEM1 knockdown effect on G0/G1 phase cell promotion was caused by the low expression of miR-129-5p (Figure 5e). In other words, we conclude that PCGEM1 modulates miR-129-5p to achieve its functional effects on PCa cells.

## CDT1 was a downstream target of miR-129-5p

**Figure 6.**
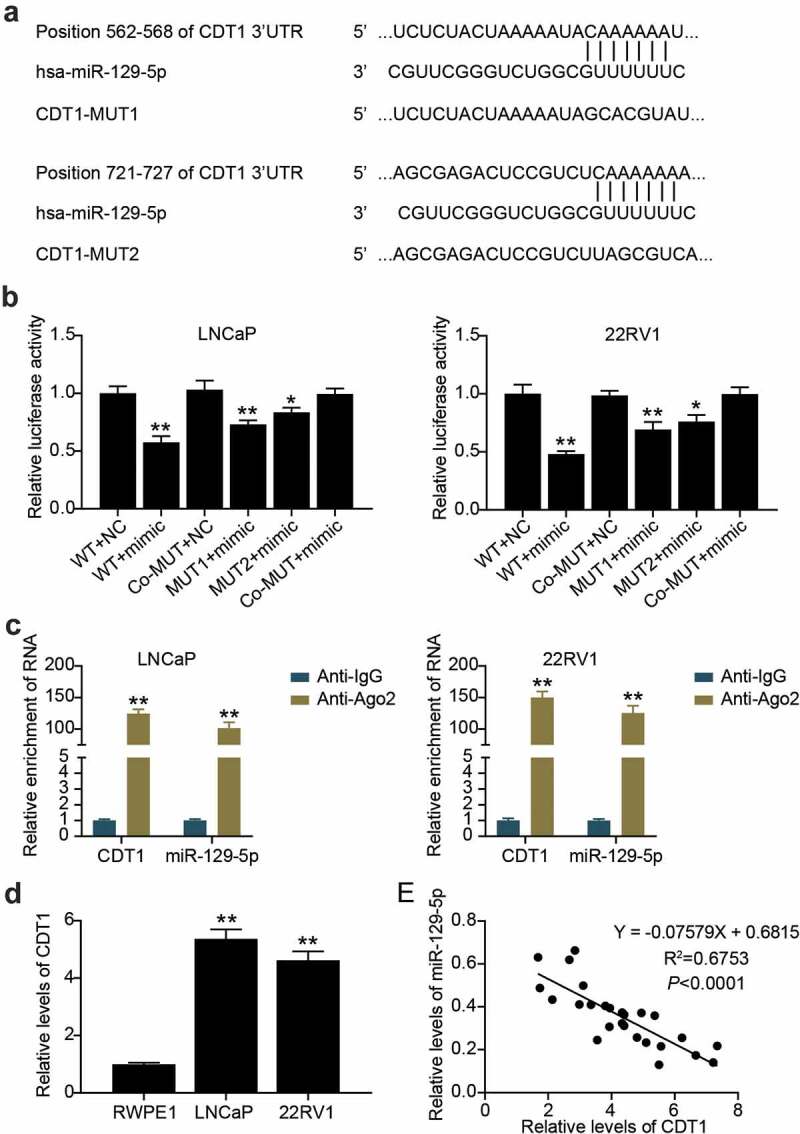
**CDT1 was a downstream target of miR-129-5p**. (a) The wild-type and mutant binding site of CDT1 for miR-129-5p were constructed. (b)The interaction between CDT1 and miR-129-5p was confirmed by luciferase reporter assay. *P < 0.05; **P < 0.001 vs. WT-NC. (c) Relationship of CDT1 and miR-129-5p was validated using RIP assays. **P < 0.001 vs. Anti-IgG. (d) The CDT1 expression in prostate cell lines was measured using qRT-PCR. **P < 0.001 vs. RWPE1. (e) Pearson was utilized for exploring the connection of CDT1 with miR-129-5p.

Targetscan predicted that there were two binding sites between CDT1 and miR-129-5p (Figure 6a). The luciferase assay showed that compared with the WT + NC group, the luciferase activity of cancer cells in WT + mimic, MUT1 + mimic, and MUT2 + mimic groups decreased by 50%, 30% and 20%, respectively (Figure 6b). In addition, the RIP assay revealed that miR-129-5p and CDT1 were enriched in Anti-Ago2 antibody-precipitated compounds compared with anti-IgG (Figure 6c). We further found that CDT1 in LNCaP and 22RV1 cells was 5 and 4.5 times higher than that in RWPE1 cells, respectively (Figure 6d). Additionally, miR-129-5p levels were inversely correlated with CDT1 levels (Figure 6e). miR-129-5p targets CDT1.

## miR-129-5p restrained PCa cell survival and facilitated apoptosis by regulating CDT1

**Figure 7.**
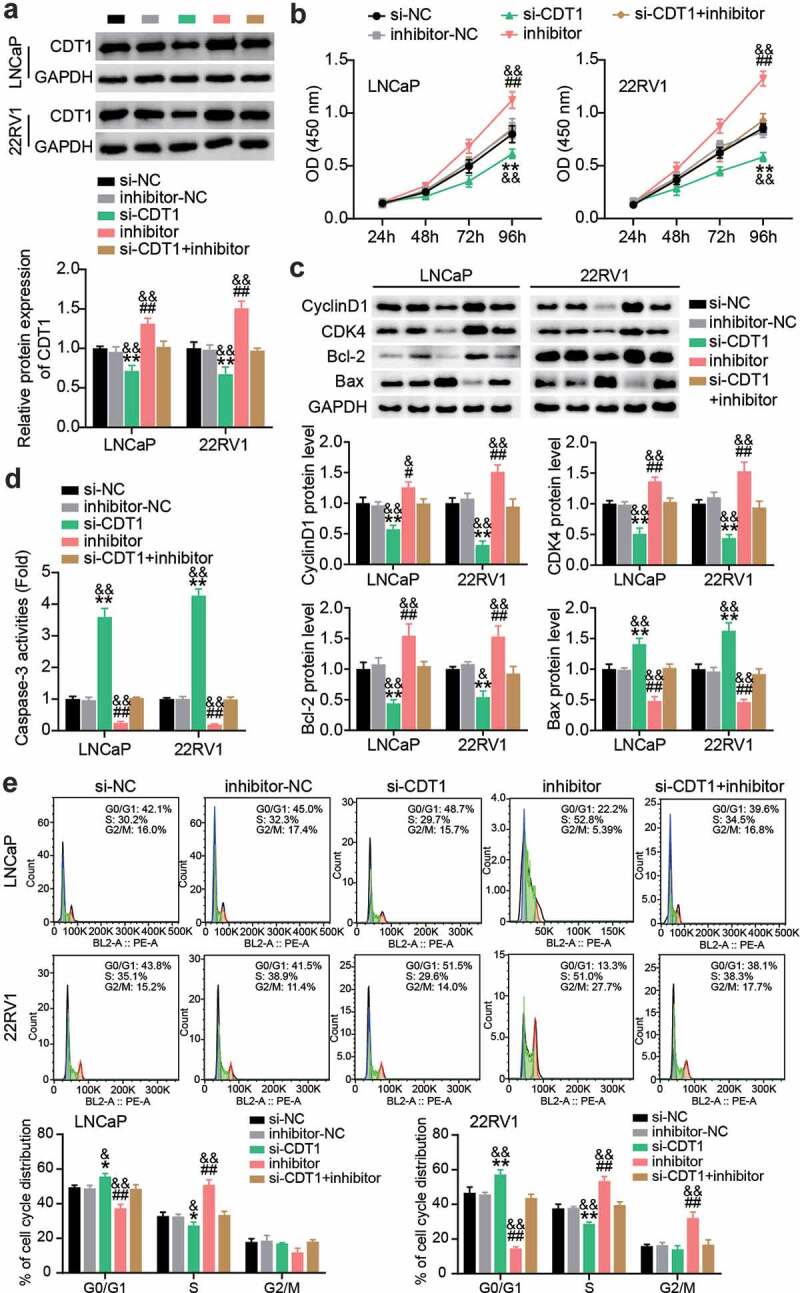
**miR-129-5p restrained PCa cells survival whereas facilitated apoptosis by regulating CDT1**. (a) The CDT1 protein expression was evaluated using western blotting assay in LNCaP and 22RV1 cells treated by si-CDT1. (b) The viability of LNCaP and 22RV1 cells treated by si-CDT1 was assessed using the CCK-8 assay. (c) Bax, Bcl-2, CyclinD1 and CDK4 protein levels were evaluated using western blotting assay in LNCaP and 22RV1 cells treated by si-CDT1 inhibitor. (d) The caspase-3 activity of LNCaP and 22RV1 delivered si-CDT1 was uncovered utilizing the caspase-3 activity assay. (e) Cell cycle of LNCaP as well as 22RV1 delivered si-CDT1 was assessed using flow cytometry assay. *P < 0.05, **P < 0.001 vs. si-NC; #P < 0.05, ##P < 0.001 vs. inhibitor-NC; &P < 0.05, &&P < 0.001 vs. si-CDT1+ inhibitor.

siRNAs targeting CDT1 and miR-129-5p inhibitor were transfected into LNCaP and 22RV1 cells. Western blotting showed that miR-129-5p inhibitor treatment upregulated CDT1 protein levels, while si-CDT1 treatment downregulated CDT1 protein levels (Figure 7a). The CCK-8 assay revealed that the viability of LNCaP and 22RV1 cells was significantly inhibited after CDT1 knockdown, and the pro-cell viability effect of miR-129-5p inhibitor was largely mitigated (Figure 7b). In addition, western blotting showed that CDT1 knock-down inhibited CyclinD1, CDK4, and Bcl-2 protein levels; promoted Bax; and reversed the regulatory effect of miR-129-5p inhibitor (Figure 7c). Caspase-3 activity analysis showed that the level of apoptosis was increased after si-CDT1 transfection, and the inhibition of apoptosis by miR-129-5p inhibitor was eliminated (Figure 7d). In addition, after downregulation of CDT1, the proportion of G0/G1 cells increased, and the proportion of S decreased, while knockdown of miR-129-5p reversed this effect (Figure 7e).

## Discussion

lncRNAs play a crucial role in multiple processes that modulate gene expression, and their transcription can lead to gene silencing or gene activation [29,30]. It has been revealed that lncRNAs participate in the biological processes of many diseases, including cancer, regulating cell proliferation, apoptosis, differentiation, and metastasis [31,32]. For example, elevated levels of lncRNA ROR in PCa tissue are associated with late tumor staging, lymph node or distant metastasis, and modulation of the AKT pathway to promote malignant proliferation of PCa cells [33]. Overexpression of lncRNA PVT1 in PCa tissues and cells is associated with low overall and disease-free survival and tumor staging [34]. Down-regulated lncRNA CRNDE inhibits the proliferation and migration of PCa cells and induces apoptosis [28].

Although many lncRNAs have been discovered, their exact functions and potential mechanisms in cancer remain to be further investigated. Here, we investigated the role of PCGEM1 in PCa. PCGEM1 was found to be upregulated in PCa, which was similar to previous studies [35]. In addition, in previous studies, the silencing of PCGME1 by small interfering RNA significantly restrained PCa cell proliferation and growth, and induced early apoptosis [36,37]. Based on these results, we further found that knockdown of PCGME1 restrained the viability of PCa cells and resulted in cell cycle arrest. In addition, subcellular localization experiments showed that PCGME1 plays a role in the cytoplasm of PCa cells.

IncRNAs can exert miRNA sponge effects through the ceRNA mechanism [38]. In this study, we found that miR-129-5p was negatively regulated by PCGEM1 and negatively correlated with PCGEM1 in PCa. Using a bioinformatics website and luciferase assay, PCGEM1 was revealed as a sponge of miR-129-5p. In addition, RNA immunoprecipitation with Ago2 revealed that miR-129-5p and PCGEM1 were highly enriched in PCa cells. This result is consistent with the findings of Zhang et al. [14] and Li et al. [15] that PCGEM1 targets and negatively regulates miR-129-5p. Additionally, we further revealed that miR-129-5p was under-expressed in PCa, and miR-129-5p knockdown promoted cancer cell viability and inhibited apoptosis and cycle arrest. This is consistent with previous studies that have reported miR-129-5p as an anticancer agent in PCa [13].

DNA replication licensing factor CDT1, a basic protein that replicates the corresponding origin licensing in G1, is degraded in the S phase and reaccumulates in the G2 phase [39]. The accumulation of CDT1 regulates the DNA replication phenotype, thus affecting tumor cell replication and apoptosis [40]. Accumulated literature suggests that CDT1 may be a new marker for cancer diagnosis and prognosis [41]. CDT1 was upregulated in both hepatocellular carcinoma [17] and breast cancer [18]. However, in a study of lung cancer, CDT1 expression was shown to be suppressed in cancer tissues and cells [42]. It was speculated that the differential expression of CDT1 may be related to the tissue type. In this study, we uncovered for the first time that CDT1 was expressed more in PCa, and its knockdown restrained LNCaP and 22RV1 cell viability. This conclusion was similar to that found by Takeo et al. [19] in LNCaP cells with increased proliferation and increased CDT1 expression. In addition, this study found that silencing CDT1 promoted apoptosis and cell cycle arrest in PCa cells, which had similar properties to the initiation of CDT1 replication in G1 phase cells in yeast strains [43]. Furthermore, we found that CDT1 was the downstream target gene of miR-129-5p, and CDT1 knockdown eliminated the effect of interfering miR-129-5p on PCa cells.

Although solid studies have been conducted on the role of PCGEM1/miR-129-5p/CDT1 in PCa, there are still limitations to our study. On the one hand, we did not study the effect of PCGEM1 on tumor metastasis *in vivo*. In addition, the correlation between PCGEM1 and survival prognosis and cancer metastasis in PCa patients requires further study.

## Conclusion

Our study showed that PCGEM1 promoted the progression of PCa by acting on miR-129-5p and modulating CDT1 through sponges. It is suggested that PCGEM1/miR-129-5p/CDT1 could be used as a biomarker for PCa screening and as a therapeutic target.

## Supplementary Material

Supplemental Material

## Data Availability

The datasets used and/or analyzed in this study are available from the corresponding author on reasonable request.

## Code availability

Not available.
