# Supplementary material for: Long non-coding RNA-PCGEM1 contributes to prostate cancer progression by sponging microRNA miR-129-5p to enhance chromatin licensing and DNA replication factor 1 expression
Source: Bioengineered. 2022 Apr 12;13(4):9411–24. doi: 10.1080/21655979.2022.2059936 (PMC9162030; doi:10.1080/21655979.2022.2059936)
Supplement: Supplemental Material [file KBIE_A_2059936_SM8990.zip › supplementary/Supplementary Table 1_revised.docx]

Supplementary Table 1. The clinical characteristics of PCa patients.

| Variables | N (%) |
| --- | --- |
| Age |  |
| ≤60 | 17 (65.38%) |
| >60 | 9 (34.62%) |
| Smoking status |  |
| No | 12 (46.15%) |
| Yes | 14 (53.85%) |
| Gleason score |  |
| ≤7 | 19 (73.08%) |
| >7 | 7 (26.92%) |
| Serum PSA (ng/ml) |  |
| ≤20 | 12 (46.15%) |
| >20 | 14 (53.85%) |
| Resection margin |  |
| Negative | 15 (57.69%) |
| Positive | 11 (42.31%) |
| Nodal stage |  |
| pN0 | 16 (61.54%) |
| pN+ | 10 (38.46%) |
